# Experiences of medical device innovators as they navigate the regulatory system in Uganda

**DOI:** 10.3389/fmedt.2023.1162174

**Published:** 2023-04-27

**Authors:** Brenda T. Nakandi, Owen Muhimbise, Ashley Djuhadi, Martha Mulerwa, Janet McGrath, Philippa Ngaju Makobore, Andrew M. Rollins, Robert T. Ssekitoleko

**Affiliations:** ^1^ Biomedical Engineering Unit, Department of Physiology, School of Biomedical Sciences, College of Health Sciences, Makerere University, Kampala, Uganda; ^2^ Department of Macromolecular Science and Engineering, Case Western Reserve University, Cleveland, OH, United States; ^3^ Instrumentation Division, Uganda Industrial Research Institute, Kampala, Uganda; ^4^ Department of Anthropology, Case Western Reserve University, Cleveland, OH, United States; ^5^ Department of Biomedical Engineering, Case Western Reserve University, Cleveland, OH, United States

**Keywords:** medical devices regulation, regulations landscape in Uganda, investigational medical devices, ideation through market readiness, medical device clinical trials

## Abstract

**Objective:**

A medical device must undergo rigorous regulatory processes to verify its safety and effectiveness while in use. In low-and middle-income countries like Uganda however, medical device innovators and designers face challenges around bringing a device from ideation to being market-ready. This is mainly attributed to a lack of clear regulatory procedures among other factors. In this paper, we illustrate the current landscape of investigational medical devices regulation in Uganda.

**Methods:**

Information about the different bodies involved in regulation of medical devices in Uganda was obtained online. Nine medical device teams whose devices have gone through the Ugandan regulatory system were interviewed to gain insights into their experiences with the regulatory system. Interviews focused on the challenges they faced, how they navigated them, and factors that supported their progress towards putting their devices on the market.

**Results:**

We identified different bodies that are part of the stepwise regulatory pathway of investigational medical devices in Uganda and roles played by each in the regulatory process. Experiences of the medical device teams collected showed that navigation through the regulatory system was different for each team and progress towards market readiness was fuelled by funding, simplicity of device, and mentorship.

**Conclusion:**

Medical devices regulation exists in Uganda but is characterised by a landscape that is still in development which thereby affects the progress of investigational medical devices.

## Introduction

Medical devices play an essential role in clinical decision-making processes and improvement of patient outcomes (1, 2). This contributes to achieving key performance indicators and targets such as in the United Nations Sustainable Development Goal of good health and well-being (SDG3) (3). However, healthcare facilities in low- and middle-income countries (LMICs) have limited access to functional essential medical devices (4, 5). A study assessing access to essential technologies for safe childbirth in LMICs showed that 40% of medical equipment were non-functional compared to high-income countries (HICs), with less than 1% non-functional medical equipment (6). This disparity stems from a reliance on imported equipment in LMICs hospitals—80% of which is donated (7)—and insufficient training for proper use and maintenance (8–10). In turn, this renders equipment unreliable to provide timely diagnosis, prevention, monitoring, and treatment of disease, thus accentuating inequities in overall health outcomes (2), which contribute to the high mortality rates in LMICs (11, 12).

This gap can be tackled, in part, by developing internal systems in LMICs in order to decrease the dependence on imported medical technology, especially during grim situations such as COVID 19. When COVID-19 was announced as a global pandemic in March 2020 (13), several HICs banned export of critical care medical equipment such as mechanical ventilators and patient monitors (14), leaving many LMICs that rely heavily on imported medical devices unable to manage critical COVID-19 patients adequately.

While COVID-19 exacerbated the consequences of reliance on imported medical devices, inadequate access to medical devices in LMICs is not a new issue has continually attracted interest on global health platforms (15). Some scholars suggest the design of frugal but appropriate devices as the most optimal solution to this pressing need (16). In Uganda, efforts in this direction are starting to show promise. An increasing number of innovations developed locally, sometimes as part of international teams are emerging (17), signalling a possible shift from overdependence on health technology from HICs. This is driven in part by an increase in Ugandan institutions training biomedical engineers, targeted funding from both international organisations such as the UK Medical Research Council (18) and Grand challenges Canada (19), as well as local funders such as Makerere University Research Innovation Fund (20, 21) and the Ugandan Ministry of Science & Technology (22).

In Uganda to date, only low-risk, locally-manufactured devices like incinerators (23) have reached market readiness. One of the leading causes for this is the lack of a clear regulatory framework and pathway for investigational medical devices (IMDs). According to the World Health Organisation (WHO), a clear and robust regulatory framework is necessary to guarantee public health, safety, and performance of imported medical devices and those locally manufactured (24). Although there is a presence of national regulatory bodies for medicines and health products in most African countries (25), a review of regulatory bodies for ten African countries including Uganda (26), found that these bodies are primarily concerned with regulation of imported medical devices and medicines, as opposed to locally made devices. The National Drug Authority (NDA), a Ugandan regulatory body for example, has elaborate guidelines for introducing new locally manufactured pharmaceutical products onto the market that detail requirements for production, testing, packaging, and licensing (27).

Due to uncertainties regarding how to navigate the regulatory system, innovators who seek approval for their IMDs in Uganda tend to follow more established regulatory systems such as the USA Food & Drug Administration's (FDA) Centre for Devices & Radiological Health Regulations or the European Union (EU) CE marking regulations. NDA allows for products that are already approved from recognized reference markets (such as in International Medical Device Regulators Forum countries) to leverage these approvals *via* an expedited review in Uganda. Under this arrangement, a local manufactured IMD that has obtained such approvals can be licensed (28). However this comes with drawbacks (29) such as prohibitively expensive costs related to ensuring compliance and application fees (30), and limited knowledge within the local innovator community of navigating these foreign regulatory systems.

For devices that do not have any foreign approvals, the pathway to approval in Ugandan markets is not well known. Thus, the aim of this paper is to explore, understand, and evaluate the existing Ugandan medical device regulatory systems by interviewing local IMD innovators that have navigated the system and approval process.

## Methodology

The material for this report was collected primarily from employing two sources: (1) Review of public information regarding device regulation and (2) innovators' reports of their experiences with developing new medical devices in Uganda.

Sources of information about medical regulation: A search was undertaken to find publicly accessible information about medical device regulation in Uganda. Specific websites from relevant bodies such as the Uganda Ministry of Health, Uganda National Bureau of Standards (UNBS), and NDA, among others, were examined**.** From these sites, we were able to conduct background research on all existing regulatory bodies in Uganda to establish the roles played by each body in the regulatory landscape.

Innovators: Technologies at various stages along the pathway to market readiness were identified from the networks of the authors. The criteria for inclusion in this discussion were:

• A device solving a *local* health care challenge, and developed for the Ugandan market, regardless of the origin or nationality of innovators or manufacture;
• Innovators that have attempted to go through Uganda's regulatory process by interacting with the Ugandan bodies involved in the process.

Innovators who have experience with getting approval for a device to fit the description of an investigative medical device (IMD) (31) through the current regulatory system shared:

• experiences with the stepwise process each innovator went through as part of regulation of their IMD;
• information about resources such as mentorship and funding support that proved helpful in navigating the process;
• experiences while developing documentation and material required at each step of the regulatory process;
• the challenges of navigating and lessons learned from meeting the regulatory requirements of the Ugandan system.

### Data collection

A series of semi-structured interviews were conducted face-to-face and virtually between February 2020 and March 2021, involving eight participants representing nine projects, of which seven were started by Ugandans, one by Australians, and one by Americans.

An interview guide was developed to collect each participant's experiences working within the regulatory system. Topics included: the resources and approvals required for acquiring user-needs validation, technical development of their product, and their pre-clinical and clinical trials, as appropriate. At each stage, we gathered information about the key players (i.e., innovation hubs and academic/clinical mentors) and what factors contributed to a momentum through the process and conversely, which factors hindered progress in the process.

## Results

We identified nine projects that covered a broad spectrum of areas including diagnosis, therapeutics and monitoring Each team, as shared by interviewees, composed of at least 3 members from different professional backgrounds including engineering, medical fields, and business management. None of the teams interviewed had progressed to market approval, so we could only analyse the stages prior to market approval. Table 1 below lists the teams interviewed, their affiliations, year founded, country of origin, the problem being solved, and proposed solution.

**Table 1:** Description of the medical device teams.

Team	Problem being solved	Product	Country of Origin	Affiliations^a^	Founded
EcoSmart	Inadequate supply of menstrual products	Non-reusable Pads	Uganda	MUST, CAMTech, Kao Corporation, Outbox	2016
FreO_2_	Inadequate supply of oxygen in hospitals	Low pressure oxygen plant	Australia	RAN, USADF University of Melbourne	2010
Instrumentation Unit, Technology Development Centre	Challenges with patient safety and delivering a uniform flow rate for intravenous therapy in adults and children	Electronically controlled gravity feed infusion set (ECGF)	Uganda	UIRI, DwB, Fraunhofer institute	2014
Mama Ope	Diagnosis of pneumonia	Biomedical smart jacket	Uganda	MAK, RAN, Villgro	2016
Moyo	Diagnosis of preeclampsia	Preeclampsia test strip	Uganda	MAK, Duke University	2014
Neopenda	Difficulty in diagnosing when a baby was in distress	Vital signs monitor	USA	MAK, Villgro, Columbia University, Tufts Medical Centre	2015
Principality Medtech	Postpartum Haemorrhage (PPH)	PPH Belt	Uganda	MAK, RAN	2015
Shishi International	Need to share oxygen from the same source and pricey oxygen splitters. Absence of eye shields in hospitals	Oxygen splitters and phototherapy eye shields	Uganda	University of Maryland	2016
Wekebere	Difficulty in diagnosis of foetal vital signs	Vital signs monitor for expectant mothers	Uganda	UIRI, RAN	2015

^a^ Acronyms of the Affiliations: Mbarara University of Science and Technology (MUST); Makerere University (MAK); Outbox, Resilient Africa Network (RAN), and CAMTech are innovator hubs; Uganda Industrial Research Institute (UIRI); School of Public Health (SPH); United States African Development Foundation (USADF); Design without Borders (DwB).

### Bodies involved in medical device regulation in Uganda

Table 2 describes the bodies involved in medical device regulation in Uganda based on available information on their websites.

**Table 2:** Descriptions of bodies involved in regulation in Uganda.

Body involved in Regulation	Role played by the body
Uganda National Bureau of Standards (UNBS)	• Formulates, enforces, and promotes standards developed with consultation from stakeholders like manufacturers, consumers, and regulators with the goal of protecting the public and environment (32–34) • Medical devices have a dedicated committee known as the Uganda National Bureau of Standards Technical Committee 14 (UNBS/TC 14) (32–34)
National Drug Authority (NDA)	• Ensures the availability of essential efficacious and cost-effective drugs. • Regulates the production, importation, exportation, marketing, and use of drugs in Uganda. • Only two medical device guidelines are listed on their website. ○ Registration of surgical instruments and appliances ○ Quality requirements for medical face masks (28). • Issues licences to suppliers for importation of medical devices. (28) • Issues licences to manufacturers intending to produce medical devices locally. ○ Only a licence and as such there is still need for product-specific guidelines for manufacturing and testing locally made medical devices. • Provides services such as online innovations and research desk that gives regulatory information regarding drugs, devices or medical products under development (27)
Uganda National Council of Science and Technology (UNCST)	• Advises and coordinates the formulation of national policies on all science and engineering fields (35) • Often the final step to approve the majority of research endeavours, including investigational medical devices studied in Uganda. • Offer mentorship by recommending bodies that help innovators implement their projects. • Gives guidance on developing IP documents before submission to the Uganda Registration Services Bureau (URSB)
Uganda Registration Services Bureau (URSB)	• Responsible for all civil registrations including utility models, patents, and other IP rights (36) • Utility models are issued for protection within the borders of Uganda. • Patents are issued in consultation with the African Regional Intellectual Property Organisation (37)
Ministry of Health (MOH)	• Directs policy and standards of healthcare in Uganda. • Oversees imported or donated medical equipment through the Health Infrastructure Department. • From interviews, learned that the Directorate of Curative Services at MOH recently mandated that they assess all investigative medical products before continuing to UNCST in response to the NDA's lack of capacity to evaluate research protocols for IMDs
Research Ethics Committees (RECs)	• 24 RECs housed in higher learning institutions and hospitals in Uganda that are officially accredited by UNCST. • Approve IMD clinical studies and focus on protecting potential study participants, society, and researchers.

### Experiences with the medical device regulatory pathway

**Figure 1:**
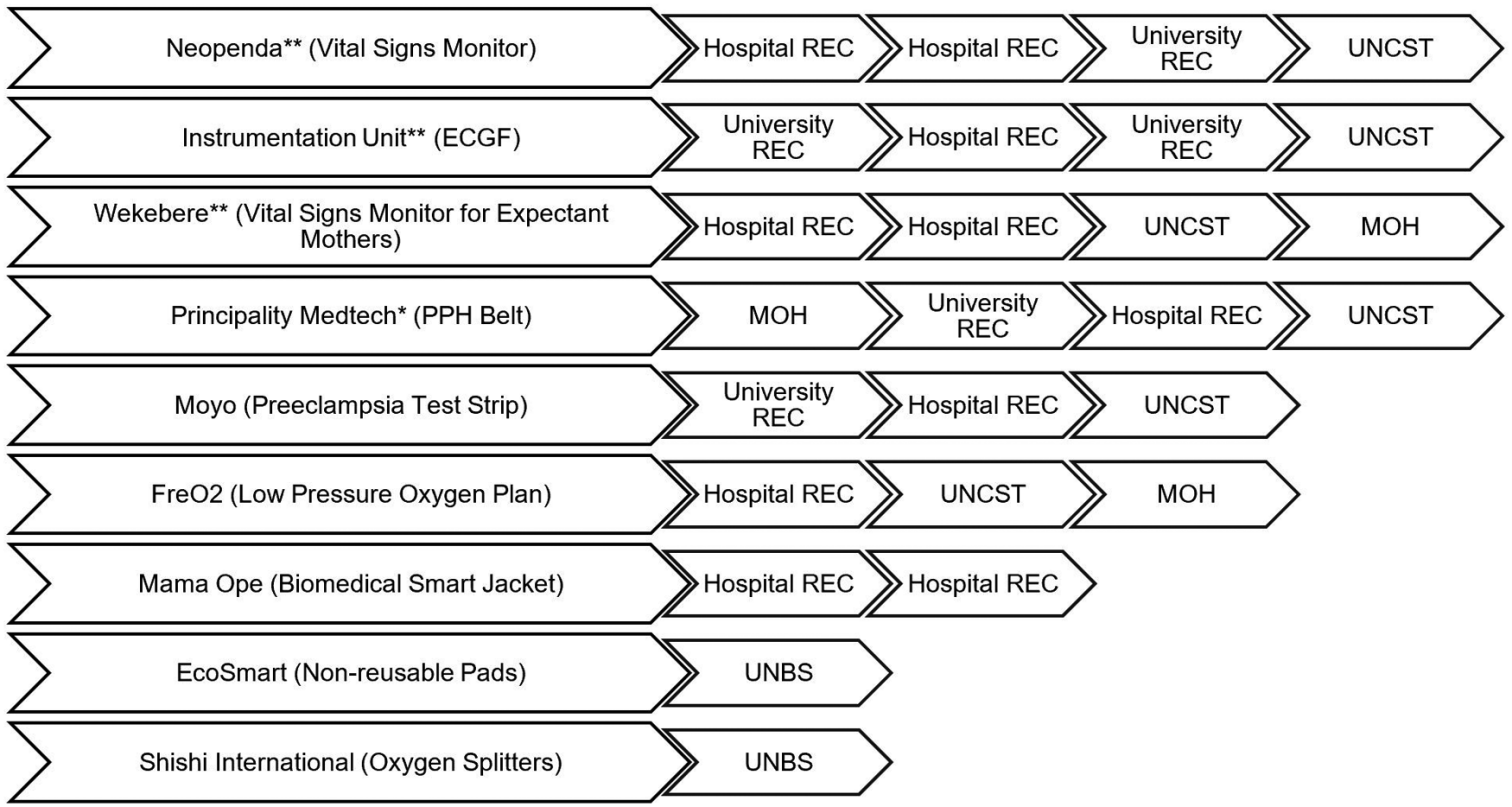
Flow chart of approvals that innovators received from regulatory bodies by company. Multiple boxes of RECs of the same kind (e.g. from hospital REC to hospital REC for one team) means that the team went to two different RECs. (*) The innovator went to the NDA first and was told NDA does not carry out IMD regulation. (**) obtained waivers from NDA stating that they lack the capacity to assess IMDs in terms of safety and performance, and recommended the entity proceeds with clinical trials provided they have approval from a REC and UNCST.

The pathway for obtaining regulatory approval in Uganda for medical technologies that have not been licensed by other markets is not straightforward and has not been formally structured. Often, innovators are advised by colleagues and mentors in business, academia, clinical practice, and relevant government institutions. The following section describes how the approval process between teams has differed as seen in Figure 1, and further stratified into general categories that were identified as common topics amongst teams. These include the clearance process as they sought human subjects research approval, innovation hubs they interacted with, funding received during the design process, and challenges experienced.

### Human subjects research approval

Researchers must submit study protocols and receive approval from a REC accredited by UNCST for studies involving human subjects (38, 39). All teams that carried out clinical testing of their devices sought clearances from at least one university REC. The specific pathways to approval varied notably.

Four teams received initial clinical study approval from one university or hospital REC. Upon clearance from these RECs, all teams except Mama Ope advanced to the UNCST for final clearance. The UNCST recently enacted an additional requirement to present to an appropriate Technical Working Group (TWG) at the MOH alongside the approval letters and proposal previously submitted to the body. Although Wekebere had to present to a TWG, Neopenda and the Instrumentation Unit received letters from the office of the Director of Curative Services to support their REC and UNCST applications without presenting to a TWG. Three other teams differed where they obtained their approval. Principality MedTech had their initial protocol assessment done by MOH through the Directorate of Curative Services, assisted by TWGs after which they got clearance that was attached to the protocol submitted to university REC and subsequently UNCST. The other teams—EcoSmart and Shishi International—had their products evaluated by UNBS only because they did not require clinical trials for their products.

### Innovation hubs

Most of the teams relied heavily on innovation hubs for support along various stages of the process. Innovation hubs (also known as incubator hubs) generally provide resources related to community entry skills, user needs evaluation, pre-clinical trials training, prototyping space, team composition mentorship, and financial support and sourcing.

For example, from Resilient Africa Network (RAN) (40), Wekebere received mentorship for writing REC documentation and lobbying for funding to pay for approval fees that enabled them to continue their project. RAN pre-negotiated approval for Mama Ope's first hospital study with Makerere School of Public Health. Mama Ope and EcoSmart, also noted that CAMTech helped them develop their clinical studies, determine the statistics for sample sizes, define criteria for studies, and forge health centre connections.

Teams founded during their undergraduate studies also attributed success to the mentorship provided by professors and clinicians they met during their coursework. These mentors guided them through the process based on their own prior experiences, which greatly aided proposal submissions to RECs, and execution of studies.

### Funding

Teams' progress was highly dependent on the availability of funds to support their efforts. While funding sources did not necessarily advise teams on steps of the regulatory process, they were a vital component for financing all of the teams' product development and regulatory journeys. Table 3 below shows grant sources attained by teams for support. Notably, teams with stable funding and solid international networks progressed to more advanced stages compared to their counterparts. For example, the Instrumentation unit had sustained funding from the ideation stage to their current stage, namely the second phase of the international patent application under the Patent Cooperation Treaty (PCT) and getting their device CE-mark ready for manufacturing. This was achieved through consultation and subcontract work with Design without Borders (DwB), a design hub. Conversely, Mama Ope halted their progress at the needs assessment stage until financial support was secured.

**Table 3:** Funding received by innovators categorized by sponsor type.

Company	University Competition	Industry/ Non Governmental Bodies	Government	Innovation Hub	Others
EcoSmart	Big Ideas	Uganda Development Bank, Kao Corporation	Up Accelerate (United Nations Populations Fund)	Not applicable	Not applicable
Mama Ope	Big Ideas	Not applicable	United States Agency for International Development (USAID)	Not applicable	Not applicable
Principality MedTech LTD	Big Ideas	Not applicable	Grand Challenges Canada	Not applicable	Not applicable
Wekebere	British Royal Academy of Engineering	Not applicable	Not applicable	Resilient Africa Network (RAN)	Not applicable
Moyo	Big Ideas, MAK Research Fund, Duke Accelerator Competition, Duke Start-Up competition, Kenan Biddle Engineering (Duke University)	Not applicable	Grand Challenges Canada	Not applicable	Not applicable
FreO2	Not applicable	Korean International Cooperation Agency, Bill and Melinda Gates Foundation	Grand Challenges Canada, United States Agency for International Development (USAID), United Kingdom Government, Government of Norway	Not applicable	Not applicable
Neopenda	Not applicable	ADAP, Techstars	United States Agency for International Development (USAID)	Not applicable	Not applicable
Instrumentation Division	Not applicable	Patient Safety Movement Foundation	Grand Challenges Canada, Ugandan Government, German Federal Ministry of Education and Research	Not applicable	Not applicable
Shishi International	Not applicable	Not applicable	Not applicable	Not applicable	Sales and Profit

On the other hand, teams whose origin was not Ugandan (FreO_2_ and Neopenda from HICs) had consistent funding to enable them to progress at each stage of regulation.

### Degree of inventiveness and complexity

Teams whose IMDs had predicate devices and/or existing standards with NDA or UNBS encountered a relatively smooth transition to more advanced stages compared to their counterparts. This was seen with Shishi International's and EcoSmart's technologies. For Shishi International, their phototherapy shields went straight to market without the need for approval from any regulatory body. Shishi International's oxygen splitters are currently manufactured in China. Their interaction with NDA was to verify the quality control of their goods as done with other medical-related imports in Uganda. Like EcoSmart, Shishi International interacted with UNBS for their locally reassembled splitters.

### Shared challenges through the regulatory pathways

The overarching challenge that innovators faced was the process irregularities and inefficiencies associated with obtaining approvals from the regulatory bodies.

One of the main hurdles was the absence of clear procedures at each regulatory body that resulted in participants being redirected between different offices to pursue approval to conduct their studies. For example, Neopenda, Wekebere, Principality MedTech, and the Instrumentation unit teams (Figure 1) were first directed to NDA for initial assessment, which does not possess the capacity to assess such protocols.

The source for referral varied between teams varied between teams: Wekebere was advised by the instrumentation unit at UIRI while Mama Ope followed an online document that recommended visiting NDA before conducting any pilot study.

However, after the interviews were completed, a study published online in 2023 by Mpaata et al. in conjunction with the Center for Innovation, Design, and Translational Excellence (CITE) at Makerere University generated a proposed flowchart for the translation of medical device innovations in Uganda (41). This flowchart was a result of a focus group discussion including members of the NDA, URSB, UNSB, UNCST, and volunteers from the WHO-Africa Medical Device Regulators Forum (WHO-AMDRF) (41). Mpaata et al. pathway diagram suggested prior to clinical trials, teams should meet with the NDA to classify the medical device class as A, B, C, or D based on risk (41). It is important to highlight that this meeting is to classify the device (41) as opposed to first seeking approval for a clinical trial, which is what the four teams had initially done. If a clinical trial was required, it was recommended that an authorisation letter be obtained from the MOH followed by one from the appropriate REC at the selected study site, which is the path that Principality Medtech followed. All other interviewed teams went to a REC before MOH if a clinical trial was needed.

When a particular health centre was chosen as a study site, our interviews illuminated that the process to obtain approval to carry out the study was neither direct nor clear. For example, Wekebere was required to seek approval from the district local government because their intended district level health centre IV study site did not have its own REC committee. Similarly, UIRI did a study at Fort Portal Regional Referral Hospital only after getting clearance from Mbarara Regional Referral Hospital, a similar hospital in terms of hierarchy.

Even with the appropriate identification of the regulatory body, the expertise on these boards like RECs did not always have the capacity to review these technologies. Generally, these hospital/medical university RECs are composed of medical doctors of different disciplines. A study carried out by Ainembabazi et al. (42) on competencies of REC members in Uganda showed that none of the 55 were biomedical engineering professionals (42).

Additionally, there were delays in communicating feedback from these bodies to different teams' applications. For Neopenda, one stage took about nine months to receive a rejection, and in total, their timeline for approvals took about 4 years. Being the first IMD study to be presented to MAK School of Medicine (SOM) REC in 7 years at that time, the Instrumentation unit encountered numerous delays pertaining to first-time IMD approval experience, including a requirement to submit two protocols; one initial safety and efficacy pilot study involving adults and another for subsequent comparative studies in children.

Finally, Mpaata et al. suggested that innovators should submit a marketing authorisation to the NDA that then is reviewed under three different tracks depending if prior approval had been obtained internationally or not to finally receiving marketing authorization and registration to all for marketing entry with operation licences (41).

### Material required for submission of application

Another challenge was carrying out the studies to generate the data needed to apply to the regulatory bodies for market approval. This multifaceted hurdle has three major obstacles: availability of standards, access to prototyping resources, and clarity of necessary tests. This was mostly experienced at the pre-clinical validation stage, as illustrated in the following examples.

First, international standards can be cost-prohibitive, and a comparable local standard at the UNBS may not exist. Mama Ope faced this challenge during benchmarking in preparation for pre-clinical tests at a time when the project was low on funding.

Secondly, some teams could not perform prototype validation testing as UNBS did not have the required equipment. Wekebere encountered this obstacle during their pre-clinical validation stage, while Shishi International encountered it at the market-readiness stage of their fully developed oxygen splitters reassembled in Uganda. In the same vein, there is a lack of certified manufacturing facilities to produce a high volume of minimum viable products for clinical trials testing, which is essential for generating data to submit applications to regulatory bodies and gauge prototype success.

The third obstacle was a lack of clarity on required technical evaluations of products that did not have an existing predicate device on the global market. Mama Ope faced this challenge. While other wearable devices that aid in the diagnosis of pneumonia exist, there was no single standard that perfectly embodied their product. The team noted that they could not find a single standard that encompassed their entire design and as such, had to review multiple standards and scientific papers to determine a pathway for their device. Mama Ope developed their own simulations after receiving clinical data from a regional referral hospital to test their device alongside the input of doctors, consultants, and other technical experts.

## Discussion

The results of our investigation illustrate a nascent medical devices regulatory landscape in Uganda. There are multiple institutions with regulatory oversight over the development and importation of medical devices in the country. However, experiences of medical technology innovation teams demonstrate a lack of clarity in the roles of these regulatory bodies, and in some cases, overlapping mandates. Because Uganda has yet to establish a formalised regulatory pathway for biomedical products, the guidance the teams received did not always recommend the same path. All teams identified that the lack of public information about which regulatory body to approach first was a significant obstacle in navigating the regulatory pathway in Uganda. Some bodies did not clearly state their roles or had different sources providing contradictory information, which contributed to the confusion. As is common in sub-Saharan African countries (43, 44), Uganda has established basic regulatory systems that lack the capacity to execute higher-end medical device approvals. WHO recommends that local innovators need to be informed and educated about the regulatory system and its requirements (45). This can be incorporated in a formalised pathway for the regulation of IMDs in Uganda. Such a pathway may best be developed using a multi-sector approach whereby academic institutions, the health sector, and regulatory bodies within the country partner to develop the regulatory pathway.

Alternatively, Uganda could take the approach of the USA whereby regulation of medical devices (both imported and locally manufactured) is entirely administered by one body, the FDA. The FDA issues different types of approval for medical devices depending on the level of risk to the users (46). Manufacturers of low risk or Class I devices must self-certify and register their device on the FDA website before launch to market. Authorisation to market medium risk or Class II devices is issued by the FDA in form of a 510 (k) premarket notification and this is granted after submission of an application that evidences that a device is safe and effective (46). The application is required to demonstrate significant equivalence to a predicate device, already on the market (46). Class III devices or high-risk devices undergo the most stringent scrutiny. Manufacturers are required to participate in pre-approval audit of manufacturing facilities and Quality Management Systems with the FDA. They must submit documentation of results on non-clinical trials, clinical trials, safety, and effectiveness data. Before a prerequisite clinical trial is carried out, an Investigational Device Exemption (IDE) must be sought from the FDA (46). At the end of these processes the FDA issues Premarket Approval for successful applications for Class III devices (46). For avoidance of lack of clarity and overlap of mandate, Uganda could take this approach whereby the National Drug Authority would fully sanction the regulation of medical devices and create regulatory pathways for medical devices that are disaggregated by classes.

Innovators identified the need for a central point to access all information about regulation similar to what is enacted by well-established bodies like the FDA in the US, the Therapeutic Goods Association in Australia, or the Therapeutic Products Directorate in Canada. According to the Ugandan Public Health Act(45) (47), the body responsible for overseeing the health delivery system in Uganda is the MOH. This paper highlights the need for focused efforts by the MOH and other regulatory bodies to develop and provide explicit and comprehensive information concerning the medical devices regulatory framework in Uganda and make it readily available for innovators and researchers to design their studies appropriately before review. This framework should be published online, distributed to common resources like innovator hubs, in addition to being incorporated into the curriculum for science and technology students. Teaching about the regulatory process within Uganda could be a complementary component of university education in translational health technologies and their applications (48).

RECs could expand their inclusion criteria to include a varied technical expertise such as biomedical engineers since there is a strong biomedical engineering program at local universities such as Makerere University (17, 20, 21). In addition to developing a formalised framework, biomedical entities, such as universities, should reach out to RECSs to provide access to appropriate expertise as needed which diversify the board's expertise to holistically evaluate applications not only from the clinical perspective, but also from a technical feasibility and design quality perspective (25) as biomedical engineers are trained to do.

Regarding the lack of standards for some IMDs, development of medical device standards is a tedious and financially demanding process. With a collaborative strategy, however, combined efforts amongst all stakeholders in the medical device design process can yield contextualised standards that are easily accessible to local innovators (45). In order to increase the ability of locally developed products to reach market readiness, the Ugandan government should be encouraged to provide funds to adopt and tailor already existing international standards like ISO 16142-2:2017 (49) for medical devices intended for the local market. This can be done along with collaboration between leading academic institutions and hospitals that can lend their expertise and insight to ensure these standards assess the safety and effectiveness of new technologies.

There are several limitations with our study. First, the scope of this study focused on interviewing the experience of innovators that attempted to seek medical device approvals in Uganda but did not include engaging representatives of said bodies that are involved in the regulation. Speaking directly to these bodies to elucidate their perspectives of the order of events to seek approval, how they use the recommendation and evaluation of other bodies when assessing proposals, and their general suggestions to innovators on how to achieve approval from their office would have greatly enhanced the study. Second, none of the innovator teams had accomplished obtaining medical device approval at the time of interviewing which limits the study by not having a complete case that has reached market entry as a model for which other innovators could follow. Third, innovators had not obtained approval for these products elsewhere in foreign markets either. And so, this study is limited to focusing on track three of medical device approval as opposed to track one and two, which is for products pre-approved in other countries (41).

## Conclusion

In conclusion, although there is some form of regulation of IMDs in Uganda, there is a lack of clarity about the steps and standards to follow while seeking approval. Therefore, this discrepancy presents a need for a formalised and established regulatory framework agreed upon by all bodies and widely understood by stakeholders involved with innovation of locally-designed medical technologies. It would be very helpful if local bodies undertook a benchmarking exercise in jurisdictions where IMDs are comprehensively regulated such as in the USA, EU or Canada. Such a framework would be an essential part of the innovation ecosystem which positively impacts the delivery of health care services in Uganda.

## Public interest summary

To understand the steps and key players involved in ensuring safety and quality of innovative medical technologies before they reach the market in Uganda, we interviewed nine medical technology teams undergoing the process. In this paper, we share their experiences, the challenges they faced, and what enabled them to overcome those challenges.

## Data Availability

The raw data supporting the conclusions of this article will be made available by the authors, without undue reservation.
